# Implementation and Performance Analysis of Constellation Dynamic Selection in Multi-Constellation RAIM

**DOI:** 10.3390/mi13091455

**Published:** 2022-09-02

**Authors:** Qian Meng, Yuan Zhuang, Shengying Li

**Affiliations:** 1 School of Instrument Science and Engineering, Southeast University, Nanjing 210096, China; 2 State Key Laboratory of Information Engineering in Surveying, Mapping and Remote Sensing, Wuhan University, Wuhan 430072, China

**Keywords:** GNSS, navigation, positioning, ARAIM, multi-constellation, integrity

## Abstract

Global navigation satellite system (GNSS) plays a crucial role in many fields, such as aerospace and transportation. Integrity is the measure of trust used in GNSS positioning especially in safety-critical applications. Advanced receiver autonomous integrity monitoring (ARAIM), taking full advantage of multi-constellation GNSS, shows huge potential to provide vertical navigation in civil aviation en route navigation and terminal approaches. However, the multi-constellation ARAIM also greatly exposes computational complexity and potential performance hazards in fault modes determination and fault-tolerant positioning. From the perspective of integrity risk control, rather than the pursuit of better positioning accuracy blindly for safety-critical applications, the concept of constellation dynamic selection is proposed and implemented in ARAIM and the performance analysis is discussed in this paper. Only the best two constellations which have the best vertical geometry performance are involved in ARAIM calculation anytime anywhere. The proposed method shows superiority in both integrity availability and computational complexity in both simulations and actual GNSS signal experiments. While the computational complexity is less than 10% of that using four constellations, 100% availability under LPV-200 criteria can be achieved in worldwide coverage experiment. The proposed method also overcomes the shortcomings of ARAIM with two fixed constellations and shows good robustness under depleted scenarios. Furthermore, the statistics results from observation stations proved the applicability and generality of the proposed method under current developing GNSS constellations.

## 1. Introduction

Global navigation satellite system (GNSS) is one important positioning and navigation system widely used in aerial vehicles and ground transportations [1,2]. Integrity, including the ability to provide timely warnings to users when GNSS should not be used for navigation, is one of the most important indicators to evaluate and keep the performance of GNSS. Different from the Satellite-based augmentation system (SBAS) and Ground-based augmentation system (GBAS), who rely on ground stations and precision instruments to provide regional integrity information [3,4], the receiver autonomous integrity monitoring (RAIM) detects the navigation satellite fault and predicts the GNSS availability implemented on the airborne receiver. RAIM is widely used in the en route horizontal guidance for the civil aviation aircraft. Compared to SBAS and GBAS, RAIM is of special importance as it assesses the GNSS signal directly received by the aircraft [5]. In most GNSS safety-critical applications, such as autonomous vehicle and rail transportation, RAIM is also an essential function to evaluate the GNSS availability [6,7]. With the remarkable development of multiple GNSS in the past decade, i.e., Galileo navigation system created by European Union and Beidou navigation system (BDS) from China [8,9], a new generation RAIM technology called advanced receiver autonomous integrity monitoring (ARAIM) is proposed to provide integrity solution for civil aviation vertical guidance using airborne monitors. Different from the traditional RAIM, ARAIM can monitor multiple simultaneous fault modes like two satellite fault and constellation fault [10,11]. The recent Galileo constellation outage further reminds us that it definitely happened even though its probability is extremely low [12,13]. It is irresponsible to take such chances under safety-critical applications. ARAIM can effectively detect and exclude such faults and provide a higher-level integrity for GNSS availability.

The ARAIM user algorithm is implemented based on multi-constellation and dual-frequency GNSS, where the spatial geometry will be improved greatly and the ionosphere error can be corrected easily through dual-frequency observations combination [14,15]. ARAIM is outlined by the GPS Evolutionary Architecture Study (GEAS) and has been further developed within the EU-US Cooperation on Satellite Navigation Working Group C ARAIM Technical subgroup (ARAIM TSG) [16]. The research of ARAIM TSG focuses on GPS/Galileo dual-constellation. It is believed that the four global constellations, GPS/GLONASS/Galileo/BDS, will provide more solutions and tap more potentials for the ARAIM development. As a navigation integrity solution, ARAIM has also shown wide-ranging applicability and potentiality in other GNSS safety-critical applications like connected car network and anti-spoofing [17,18].

ARAIM uses multi-constellation observations to improve the satellite geometry and increase redundant measurements. However, a core question is that whether more constellations are better for receiver integrity monitoring. On the one hand, the recent simulation research has shown that based on two constellations, ARAIM can cover about 90% of the world with a 99.5% availability, where the 99.5% availability herein means that the ARAIM is available during 99.5% of the time at the test point [19]. The fact is the regions cannot meet the above requirements are mainly concentrated in the polar regions where the civil aviation demand is not strong in the foreseeable future. The user algorithm used in ARAIM with two constellations is explicit and clear as the airborne receiver can monitor two kinds of fault modes only in most cases: the single satellite fault and single constellation fault. On the other hand, once three or four constellations are included in ARAIM, although the worldwide availability can reach nearly 100%, apparently the computational complexity will explode as hundreds of fault modes need to be monitored simultaneously in the airborne receiver. Not only the two fault modes mentioned above, but also the dual satellite fault, dual constellation fault and single satellite and single constellation fault will be monitored as more satellites and more constellations result in more possible fault modes. Once one fault has been detected, the fault exclusion is also difficult as there are so many potential fault modes and the computational burden will increase further as the exclusion function will result in a second layer detection test [20]. The recent research works showed that even with four constellations, the protection levels of ARAIM still cannot meet the requirements of Category I, which is the first step for precision approach [21]. The performance upper bound of ARAIM still stays at localizer precision with vertical guidance down to 200 feet altitude (LPV-200) disappointedly without any external data assistance. What’s worse, the fault detection and exclusion (FDE) threshold and probability of hazardous misleading information will enlarge due to more fault-tolerant subsets. In summary, more constellations will increase computational complexity dramatically and there has no significant performance improvements but potential hazards.

Regarding the explosive growth of monitored fault subset, many studies have recognized this problem and proposed many methods to reduce the number of monitored fault subset [22,23]. However, there is a risk that the values of protection levels and Effective Monitor Threshold (EMT) will enlarge by increasing the assigned a priori probability of the subsets that remove many satellites. Furthermore, the excessive subsets consolidation is extremely negative for the fault detection and exclusion, as too many fault modes are combined to the constellation or orbit simultaneously and it is hard to locate the fault immediately.

What cannot be ignored is that the robustness of ARAIM with two constellations is not enough. The hidden trouble is that the single constellation fault will result in a similar performance of conventional RAIM (single constellation). Then, the fault-tolerant positioning result of the single constellation subset will depend on the performance of the corresponding constellation only [24]. The worldwide availability will be badly damaged under depleted scenarios, where satellites is removed due to fault, update or in maintenance. The removed satellites will affect the geometry of the corresponding constellation. Then the impact will pass to the single constellation subset. The recent studies have verified that the depleted scenarios would hurt the ARAIM availability seriously [25,26]. Although some response solutions are proposed, these methods mostly sacrifice the other performance like vertical positioning accuracy and cannot make up the loss completely [27,28].

To evade the performance uncertainty and the computational complexity of ARAIM with three or four constellations and improve the robustness of ARAIM with two constellations, the concept of constellation dynamic selection is proposed and implemented for ARAIM user algorithm. Only the two constellations with the best geometry will be used for ARAIM estimation. The proposed method can get an encouraging high worldwide availability with a low computation burden. Facing satellite outage or even constellation outage, the method also shows superiority in robustness. Most importantly, the flourishing development of multi-constellation provides solid foundation for constellation dynamic selection. The worldwide coverage experiment, depleted scenarios test and observation station statistics fully verified the effectiveness of the proposed method.

The rest of the paper is organized as follows: Section 2 discusses the baseline ARAIM user algorithm, including the computational complexity and potential performance hazards with multi-constellation. Section 3 analyzes the dynamic performance differences of four GNSS constellations and proposes the ARAIM method with constellation dynamic selection. Experiment test and analysis are given in Section 4. Finally, Section 5 is the conclusion.

## 2. Problem Statement

In the milestone reports of ARAIM TSG, the roadmap of ARAIM development is divide into two steps: Horizontal ARAIM and Vertical ARAIM. The Horizontal ARAIM can be considered as the solution of transitional stage from traditional RAIM to ARAIM, which aims at providing horizontal navigation for en route and terminal approach. The Vertical ARAIM aims at providing LPV-200 for worldwide aircraft landing navigation. The LPV-200 is the last period in the non-precision approach and after that is the precision approach Category I. As the Vertical ARAIM can be considered as the ultimate goal of ARAIM application, we will only consider Vertical ARAIM in this paper (hereinafter referred to as ARAIM) and the ARAIM availability criteria is seen as LPV-200.

The baseline ARAIM user algorithm is based on snapshot multiple hypothesis solution separation (MHSS). Multiple hypothesis determines the fault modes that need to be monitored to meet the integrity risk with the assist of integrity support message (ISM). Solution separation tests the consistency between the present all-in-view position solution and the fault-tolerant position corresponding to the fault modes determined in multiple hypothesis and test the differences to prompt whether a fault exists. Finally, the LPV-200 criteria, including horizontal protection level (HPL), vertical protection level (VPL), EMT, and vertical accuracy, will be used to evaluate the ARAIM performance.

The number of subsets and corresponding fault modes probabilities are calculated within the satellite prior fault probability and constellation prior fault probability which is provided by the ISM. However, ISM does not specify which fault modes need to be monitored. This determination must be made by the receiver based on the contents of ISM and the positioning resolution. Different from the traditional RAIM, which can only monitor a single satellite fault, the constellation faults and multiple simultaneous satellite fault can be detected with the assist of ISM. In ARAIM, the constellation faults and satellite faults are independent events in fault modes determination as described in:

(1) $$\begin{array}{l} P_{event,i}=P_{sat,i}\\ P_{event,Nsat+j}=P_{const,j} \end{array}$$

where *P_sat_*_,*i*_ and *P_const_*_,*j*_ represent the *i*th satellite and *j*th constellation in ARAIM, respectively, and *N_sat_* is the number of satellites in the positioning calculation.

A satellite fault is a signal in space fault state which may be caused by erroneous satellite navigation data or anomalous satellite payload events. The constellation fault is a common cause which may originate at the constellation service provides (CSPs) ground segment. They are independent events in terms of prior fault probability in the content of ISM. A fault mode is a combination of events. With the prior fault probability received from the ISM, the probability of different fault modes *p_faultmode_* can be calculated as:

(2) $$p_{fault}=\prod_{i\in idx}^{}\left(P_{event,i}\right)\prod_{j\notin idx}^{}\left(1-P_{event,j}\right)$$

where *idx* refers to the set of fault events.

Not all fault modes need to be monitored. ARAIM introduces *P_THRES_* as the threshold for the integrity risk coming from unmonitored faults. One fault mode corresponds to one fault subset. The ARAIM can work into the step of fault-tolerant positioning after the probability of unmonitored fault modes is less than *P_THRES_*.

### 2.1. Significant Burden in Computational Complexity

The number of fault subsets should satisfy that the actual monitored fault probabilities are not lower than the required monitored fault probability. The relationship can be given as the following inequality:

(3) $$P_{fault,actual}>P_{fault,req}$$

where the actual monitored fault probabilities *P_fault_*_,*actual*_ and the required monitored fault probabilities *P_fault_*_,*req*_ is calculated as:

(4) $$P_{fault,actual}=\sum_{k=1}^{N_{faultmodes}}p_{fault,k}=\sum_{k=1}^{N_{faultmodes}}\left(\prod_{i\in idx}^{}\left(P_{event,i}\right)\prod_{j\notin idx}^{}\left(1-P_{event,j}\right)\right)$$

(5) $$P_{fault,req}=1-p_{no\_fault}-P_{THRES}$$

where *p_fault_*_,*k*_ refers to the probability of *k*th fault mode. *P_THRES_* is the threshold for the integrity risk coming from unmonitored faults. *N_faultmodes_* is the total number of fault modes. *p_no_fault_* is the probability of all-in-view subset, which means no fault exists:

(6) $$p_{no\_fault}=\prod_{k=1}^{\left(Nsat+Nconst\right)}\left(1-P_{event,k}\right)$$

where (*N_sat_* + *N_const_*) represents the total number of fault events.

**Figure 1:**
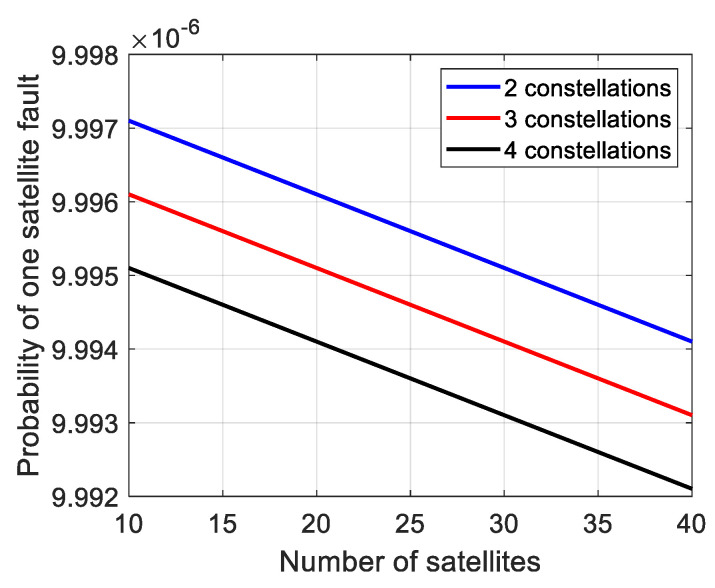
Probability of one satellite fault.

**Figure 2:**
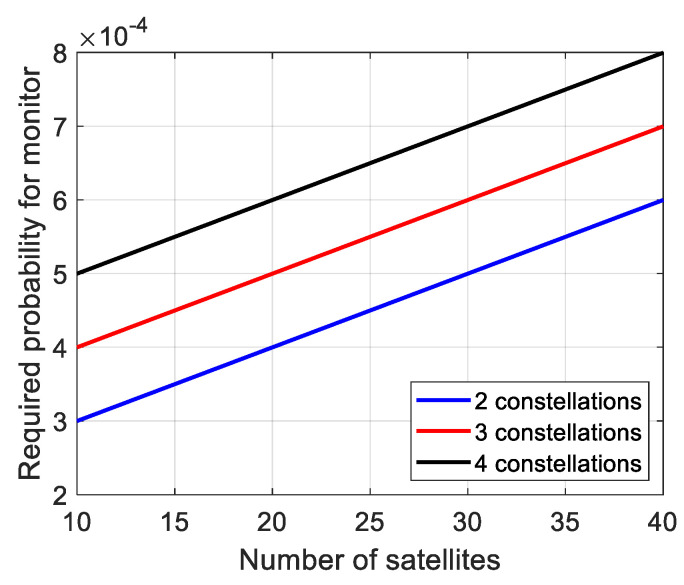
Required probability for monitor.

According to Equation (6), it is easy to understand that with more constellations involved in ARAIM, the value of *p_no_fault_* will get smaller as more fault events are involved. It will result in a bigger *P_fault_*_,*req*_ according to Equation (5). Meanwhile, on the other side of inequality (3), the probability of original *k*th fault mode gets smaller simultaneously. Figure 1 and Figure 2 show the probability trends of two fault scenarios: One satellite fault mode and total required probability for monitor, calculated by (2) and (6), respectively. The *P_THRES_*, *P_const_*_,*j*_ and *P_sat_*_,*i*_ are set as 8 × 10^−8^, 1 × 10^−4^, and 1 × 10^−5^, respectively.

One satellite fault is the basic fault mode in ARAIM. As shown in Figure 1, the probability of one satellite fault decreases with the increase in visible satellites. What is worse, more constellations result in smaller probability. Similar probability loss can be derived in the other fault modes, like two satellites fault and one satellite and one constellation fault. On the other hand, as shown in Figure 2, with the increase in visible satellites, the total required probability for monitor increases linearly. More constellations result in higher required probability. As one falls, another rises. The result is that more fault modes are needed to be monitored and more subsets are needed to be calculated to meet the inequality (3).

The recent studies unexceptionally verified the exponential increase in fault subsets. The total number that need to be monitored can be hundreds and thousands. When the number of simultaneous faults exceeds two, another core question refers to whether it needs to categorize the multiple satellites fault to the constellation fault. This is outside the scope of this article. We do not consider the specific number, but inevitably the explosive fault modes are significant computational burdens for the airborne receiver.

There are two more potential hazards in the computation burden. The first is the integrity after fault exclusion. Once one fault is detected and the corresponding satellite(s) are excluded, the solution separation will be executed again and every new subset will calculate the fault-tolerant positioning results, which means heavy computation load. The second one is the uncertainty of constellation and satellite prior fault probability. There’s no definite conclusion about the order of magnitude of fault probabilities. In recent studies, 10^−5^, 10^−4^, and 10^−3^ are all considered [29]. However, it has a great impact on the number of subsets. The current research has shown that the bigger of the prior satellite fault probability is, the more the number of subsets. Particularly, the increase can be an order of magnitude.

### 2.2. Potential Hazards in Integrity and Availability

Multi-constellation can increase the number of visible satellites and improve the geometry. Meanwhile, the increasing number of fault subsets also increases the potential hazards in integrity and makes it difficult to detect the fault.

From the aspect of fault detection, the threshold is calculated in every fault subset. In the *k*th fault subset, the difference $\Delta {\hat{x}}^{\left(k\right)}$ between the all-in-view position solution ${\hat{x}}^{\left(0\right)}$ and the fault-tolerant position solution ${\hat{x}}^{\left(k\right)}$ will test in three components with the corresponding threshold *T_k_*_,*q*_:

(7) $$\left|{\hat{x}}_{q}^{\left(k\right)}-{\hat{x}}_{q}^{\left(0\right)}{}^{}\right|\leq T_{k,q}$$

where the subscript *q* is equal to 1, 2 and 3, which represents the east, north, and up components, respectively. The threshold is defined by:

(8) $$T_{k,q}=K_{fa,q}\sigma_{ss,q}^{\left(k\right)}$$

where $\sigma_{ss,q}^{\left(k\right)}$ is the standard deviation of the difference $\Delta {\hat{x}}^{\left(k\right)}$ under nominal conditions. *K_fa_* is the threshold of the standard normal distribution. Different components correspond to different *K_fa_*. Then,

(9) $$\left\{\begin{array}{c} K_{fa,1}=K_{fa,2}=Q^{-1}\left(\frac{P_{FA\_HOR}}{4N_{faultmodes}}\right)\\ K_{fa,3}=Q^{-1}\left(\frac{P_{FA\_VERT}}{2N_{faultmodes}}\right) \end{array}\right.$$

where *P_FA_HOR_* and *P_FA_VERT_* represent the continuity budget allocated to the horizontal and vertical mode respectively. *Q* is defined as the right-hand side cumulative distribution function of a zero-mean unit Gaussian and *Q*^−1^ is the inverse of the *Q* function, also known as the inverse of the tail probability of a zero mean unit normal distribution. The step-by-step specification about the above solution separation calculation can be found in [25].

**Figure 3:**
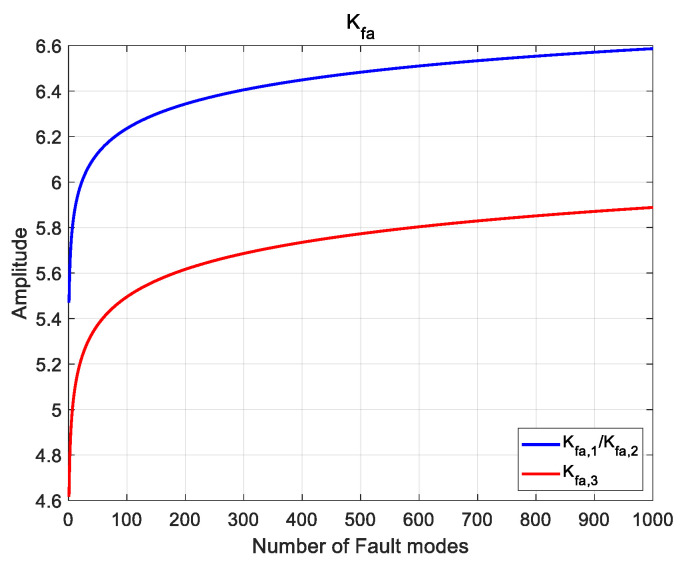
Relationship between *K_fa_* and number of fault modes.

The relationship between *K_fa_* and the number of fault modes are shown in Figure 3. With the increase in fault modes, *K_fa_* in three components will increase synchronously. It then results in the expansion of threshold. It affects the fault detection and is easy to lead to missed detection. If the number of fault modes can be limited into the interval [1~100], it can effectively control the expansion of threshold.

At the same time, the multi-constellation will affect the ARAIM availability further. EMT is one of the criteria for vertical positioning performance described by the SARPs (Standards and Recommended Practices) and accepted by the ARAIM as one of LPV-200 requirements on the vertical position. It is based on operational trials that showed that an additional requirement was needed beyond the normal requirements of vertical accuracy and integrity [14]. The EMT can be defined as the maximum of the detection thresholds of faults that have a prior equal or above *P_EMT_* (Probability used for the calculation of EMT). It is computed as follows:

(10) $$\mathrm{EMT}=\underset{k|p_{fault,k\geq P_{EMT}}}{\max}T_{k,3}=\underset{k|p_{fault,k\geq P_{EMT}}}{\max}\left(K_{fa,3}\sigma_{ss,3}^{\left(k\right)}\right)$$

where *T_k_*_,3_ is the solution separation threshold in the up component, and it is the product of *K_fa_*_,3_ and $\sigma_{ss,3}^{\left(k\right)}$. It has shown above that *K_fa_*_,3_ becomes larger with the increase of fault modes. The EMT at the current epoch will get larger and directly threaten the availability of ARAIM.

## 3. Implementation of Constellation Dynamic Selection in ARAIM

The computational complexity and performance uncertainty have been analyzed in the above section. Instead, ARAIM with two constellations is easy to implementation. However, its robustness is not good enough because the availability is seriously affected by constellation difference and satellite outages [24]. In this section, a practical ARAIM method with constellation dynamic selection is proposed. What we want to do is to select the best two constellations for ARAIM evaluation anywhere at any time. The feasibility of constellation dynamic selection is talked about first, then the particular implementation is given in details.

### 3.1. Feasibility of Constellation Dynamic Selection

The feasibility of constellation dynamic selection can be talked about from three aspects. The first is the ARAIM algorithm feasibility. The baseline ARAIM algorithm is based on snapshot principle, which means the integrity evaluation only uses the data of present epoch. There is no correlation between neighbored epochs. It provides an opportunity to select observations from different constellations to assess the integrity. The receiver will process the multi-constellation signals as usual but only the constellations selected will be transited to ARAIM evaluation.

The second aspect is the number of constellations. The satellite navigation has stepped into a new generation. All the four GNSS systems: GPS, GLONASS, Galileo, and BDS, have been providing global open service. The number of GNSS constellations supports the solution to select particular constellations to pursue high integrity while guaranteeing the positioning accuracy. In other words, it provides new ways to control the integrity risk rather than blindly pursuing the better positioning accuracy for safety-critical applications.

The third aspect of feasibility is the fact that there is performance difference among above four GNSS constellations. This difference can come from space. GPS is the only constellations which has six orbits. The orbit inclination of GLONASS is far greater than those of the other constellations. BDS accepts the mixed constellation which includes GEO, IGSO, and MEO. The above differences will result in performance differences in different places. Even in the same place, the performance difference is dynamic with the period of satellites, the satellite outages or the number of backup satellites. This difference will be aggravated by the ground characteristics when GNSS is applied to ground applications, typically the GNSS application in urban canyon. Additionally, the satellite or constellation faults will affect the performance of GNSS apparently. So, it is feasible and necessary for a receiver to select better constellations based on observations at the present moment.

### 3.2. Detailed Implementation of Constellation Dynamic Selection

**Figure 4:**
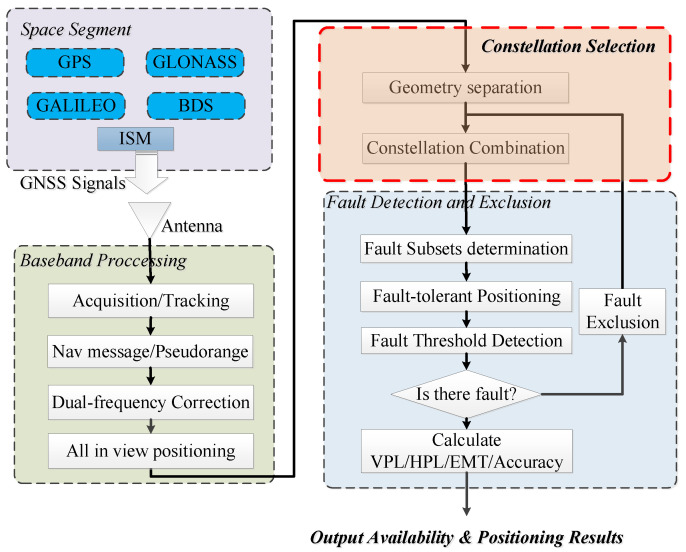
Implementation of ARAIM with constellation dynamic selection.

The detailed implementation is given in Figure 4. The implementation can be divided into three parts: Baseband processing, constellation dynamic selection and ARAIM calculation. For the baseband processing, it is a classical multi-constellation receiver including signal acquisition, channel tracking and navigation message demodulation. All the signals from four constellations are processed to get the all-in-view satellite geometry, pseudoranges, and initial positioning results. Then the constellations will be evaluated the geometry performance to get the best two constellations. Only these two constellations will be selected to assess the present integrity.

The next step is to find the criterion for constellation dynamic selection. As the basis of ARAIM user algorithm is the improved geometry from multi-constellation, the criterion should evaluate the geometry scientifically and reasonably. Furthermore, the criterion should focus on the geometry consistency at vertical component as the ARAIM focuses on providing vertical guidance. The vertical protect performance is also the biggest challenge for ARAIM application, especially the EMT requirement in LPV-200, which is also for evaluating the vertical positioning performance. Based on the research work in [24], we also noticed that in ARAIM with two constellations, the availability is related to the VDOP of spatial geometry closely. Finally, DOP values are easy to access. Considering both the performance requirements and engineering implementation, the vertical dilution of precision (VDOP) is the best choice for selecting the best two constellations. After determination of the selection criterion, the implementation can be further divided into two steps: Jacobian matrix separation and the best constellation combination selection.

#### 3.2.1. Jacobian Matrix Separation

After the signal baseband processing, the geometry matrix, also known as the Jacobian matrix has been calculated with the weighted least square calculation. The Jacobian matrix separation is given as follows.

For an epoch with *m* constellations involved, the Jacobian matrix **G** can be rewritten as:

(11) $$G=\left[\begin{array}{cc} G_{1} & T_{1}\\ G_{2} & T_{2}\\ \vdots & \vdots \\ G_{m-1} & T_{m-1}\\ G_{m} & T_{m} \end{array}\right]$$

where **G***_j_* is the line of sight (LOS) vector part of *j*th constellation and **T*_j_*** is the corresponding receiver clock offset part as follows

(12) $$G_{j}=\left[\begin{array}{ccc} x_{j}^{\left(1\right)} & y_{j}^{\left(1\right)} & z_{j}^{\left(1\right)}\\ x_{j}^{\left(2\right)} & y_{j}^{\left(2\right)} & z_{j}^{\left(2\right)}\\ \vdots & \vdots & \vdots \\ x_{j}^{\left(n\right)} & y_{j}^{\left(n\right)} & z_{j}^{\left(n\right)} \end{array}\right]$$

(13) $$T_{j}=\left[\begin{array}{ccccc} 0_{1\times n}^{1} & \cdots & 1_{1\times n}^{j} & \cdots & 0_{1\times n}^{m} \end{array}\right]$$

where $\left[\begin{array}{ccc} x_{j}^{\left(i\right)} & y_{j}^{\left(i\right)} & z_{j}^{\left(i\right)} \end{array}\right]$ is the line-of-sight vector of the *i*th satellite and *n* represents the total number of satellites in *j*th constellation. **T*_j_*** is a *n* × *m* matrix and all elements are 0 except the *j*th column, whose elements are 1.

Then the separated Jacobian matrix of *j*th constellation is given as:

(14) $$G_{j}^{\prime }=\left[\begin{array}{cc} G_{j} & 1_{1\times n}^{j} \end{array}\right]$$

#### 3.2.2. Best Constellation Combination Selection

For the best constellation combination selection, two selection rules are experimented and evaluated in this paper. Rule 1: the two constellations with the minimum VDOPs. Rule 2: the dual-constellation combination with the minimum VDOP. Corresponding to Rule 1, the constellations will be sorted by VDOP of single constellation from small to large. The top two constellations are the choices into ARAIM calculation. Then under the circumstance of Rule 2, the constellations will be combined to dual-constellation combination, only the one with the minimum VDOP is the target of constellation dynamic selection. The following part is the particular implementation of two rules in receiver algorithm.

Under Rule 1, as shown in Equation (14), it is a standard format of geometry matrix of single constellation. Then the two constellations are chosen using the following equation

(15) $${\mathrm{VDOP}}_{\min}=\min\left\{j\in \left[1,m\right]\left|\sqrt{{\left(G_{j}^{\prime }{}^{T}G_{j}^{\prime }\right)}_{}^{-1}{}_{\left(3,3\right)}}\right.\right\}$$

Under Rule 2, the Jacobian matrices of different constellations need be recombined into dual-constellation form as follows

(16) $$G_{j+k}^{}=\left[\begin{array}{cc} G_{j}^{} & T_{1}^{\prime }\\ G_{k}^{} & T_{2}^{’} \end{array}\right]$$

where $T_{1}^{\prime }$ and $T_{2}^{\prime }$ are the receiver clock offset parts, corresponding to constellation j and *k*.

(17) $$T_{1}^{\prime }=\left[\begin{array}{cc} 1_{1\times n_{j}}^{} & 0_{1\times n_{j}}^{} \end{array}\right]$$

(18) $$T_{2}^{\prime }=\left[\begin{array}{cc} 0_{1\times n_{k}}^{} & 1_{1\times n_{k}}^{} \end{array}\right]$$

where *n_j_* and *n_k_* represent the number of satellites in constellation *j* and *k*.

The number of combinations is $C_{m}^{2}$, the minimum VDOP is calculated among these combinations:

(19) $${\mathrm{VDOP}}_{\min}=\min\left\{j,k\in \left[1,m\right],j<k\left|\sqrt{{\left(G_{j+k}{}^{T}G_{j+k}\right)}_{}^{-1}{}_{\left(3,3\right)}}\right.\right\}$$

Finally, the Jacobian matrix of the selected dual-constellation combination **G***_new_* will replace the original **G** and is sent to the ARAIM part to determine the fault subsets and calculate the positioning, with the assist of ARAIM ISM information. The fault-tolerant positioning solutions ${\hat{x}}^{\left(k\right)}$ are calculated with the new Jacobian matrix as follows:

(20) $${\hat{x}}^{\left(k\right)}={\left(G_{new}W^{\left(k\right)}G_{new}\right)}^{-1}G_{new}^{T}W^{\left(k\right)}y$$

where *W*^(*k*)^ is the weighted matrix determined by the pseudorange error diagonal covariance matrices. *k* = 0 corresponds to the all-in-view positioning solution ${\hat{x}}^{\left(0\right)}$. The positioning differences between ${\hat{x}}^{\left(0\right)}$ and ${\hat{x}}^{\left(k\right)}$ will then be used for the threshold test, integrity evaluation and output the availability as mentioned in the Problem Statement.

The superiority of ‘dynamic’ is that not only the constellation selected in different epoch can be different, but also the selection in the same epoch can be different. Once one fault is detected and excluded, no matter whether it is a satellite fault or constellation fault, the program will return to constellation selection to select the best two constellations again.

## 4. Experiment and Discussion

The availability of ARAIM is the core evaluation index. In this section, the availability performance evaluation is divided into two subsections: the classical worldwide availability coverage and the particular fixed-point availability verification. The former one tests the ARAIM worldwide coverage of ARAIM 99.5% availability with the nominal constellation configurations. It is also the most frequently used for ARAIM evaluation. The latter one will fix the receiver at one particular point and collect the actual GNSS signals to assess the ARAIM availability. It aims to evaluate the ARAIM performance under the current constellations. Different ARAIM modes will be evaluated under the LPV-200 criteria.

The numerical values are given in Table 1. At the same time, as the determination of parameter values in ISM is still a major challenge before enabling ARAIM service, the choice of ISM and ARAIM integrity parameters are taken from the ARAIM TSG Milestone 3 Report and given in Table 1, too.

For the comparative test, the test objects are divided into three ARAIM modes and further refined to five groups. As shown in Table 2, three ARAIM modes are: ARAIM with two fixed constellations, ARAIM with four constellations, ARAIM with constellation dynamic selection. Then in the first model, ‘GPS + Galileo’ and ‘GPS + BDS’ are chosen as two groups in this test. The third model is also refined to two groups based on the analysis of sorting rules in the above section: the two constellations with minimum VDOPs and the dual-constellation combination with minimum VDOP.

Location points spread on a grid of 5-by-5 degree on earth and the period is one sidereal day. The time step is 600 s. Therefore, 2592 × 144 = 373,248 points are available for analysis. In turn, the main outputs of ARAIM include availability, VPL, HPL, EMT, and vertical positioning accuracy.

### 4.1. Worldwide Availability Coverage Test

The ARAIM TSG focuses on the ARAIM algorithm based on GPS and Galileo. The baseline configurations used in the milestone report have 24 GPS satellites and 24 Galileo satellites, where GPS is the 24-slot nominal constellation and Galileo is a Walker 24/3/1. It is also the nominal constellation of the above two systems. BDS MEO and GLONASS system have a similar constellation configuration to Galileo, and the nominal number of BDS MEO satellites is reduced to 24 in BDS-3 generation. The GEO satellites and IGSO satellites are not considered in this subsection as they cannot cover the whole earth. Therefore, the baseline configurations of four constellations are: 24 GPS satellites, 24 GLONASS satellites, 24 Galileo satellites, and 24 BDS MEO satellites. The above configurations are accepted in this paper to analyze the worldwide performance of ARAIM in different modes. All GNSS observations are generated using the adjusted real almanacs.

#### 4.1.1. Results under Baseline Scenario

Table 3 shows the ARAIM 99.5% availability and relative computational complexity of five groups under the LPV-200 criteria. As mentioned above, the 99.5% availability herein means that the ARAIM is available during 99.5% of the time at the test point. About the relative computational complexity, it is defined as the percentage of the computational time compared with the longest one. Here the longest one is the ARAIM with four constellations. The five groups’ tests are executed on the same software-defined receiver (SDR). The SDR runs on a mobile workstation with Intel (R) Core (TM) i7-8750H CPU @2.20 GHz and 16.00 GB RAM.

Regarding the availability, the worldwide availability of the first two groups, with two fixed constellations, can reach nearly 90% with the least complexity. The availability can be 100% after four GNSS constellation are involved. However, the computation complexity is 14 times than that of ARAIM with two fixed constellations. Focusing on the last two groups based on constellation dynamic selection, availability of both two groups using the proposed constellation dynamic selection is encouraging. The group using the two constellations with the minimum VDOP can cover 100% of the earth. The computational complexity is 7.78% of the time spent by the third group. Even compared with the first two group, the increased complexity is less than 10%. The fourth group, ARAIM using the combination of the minimum VDOP, can cover nearly 100%. However, the computational time is slightly longer than the fifth group as it need to calculate six combinations and the dimensions of matrix are also larger than those of the fifth group. In conclusion, the ARAIM using two constellations of minimum VDOP shows the best performance.

#### 4.1.2. Performance Outputs of ARAIM with Constellation Dynamic Selection

The performance outputs of ARAIM with constellation dynamic selection are analyzed and evaluated further. In particular, only the scheme that using two constellations with the minimum VDOP (corresponding to the fifth group in the above section) is considered. The outputs for LPV-200, number of fault modes and the worldwide distribution of constellation dynamic selection are shown and talked in this section.

**Figure 5:**
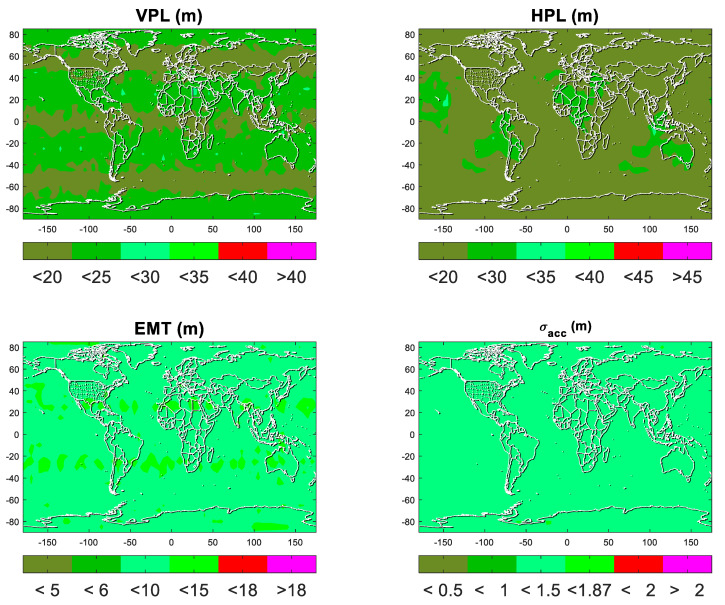
Performance of ARAIM with constellation dynamic selection. From top to bottom, from left to right is VPL, HPL, EMT, and *σ_acc_*, respectively.

Figure 5 shows the four indicators for LPV-200. The ARAIM is not considered available once any of the four indicators are not met the requirements of LPV-200. The figure shows the biggest values of 99.5% time at the test points. The plots are discretized to six color bins. Two colors of red represent values beyond the threshold and the other four colors of green represent values below the threshold. Regarding the VPL, most areas of the world are below 25 m and equatorial and mid-latitude regions are even below 20 m, which is far below the requirement of VAL = 35 m. The same phenomenon can be found in HPL, EMT, and vertical accuracy. The two constellations selected keeps the performance of ARAIM.

**Figure 6:**
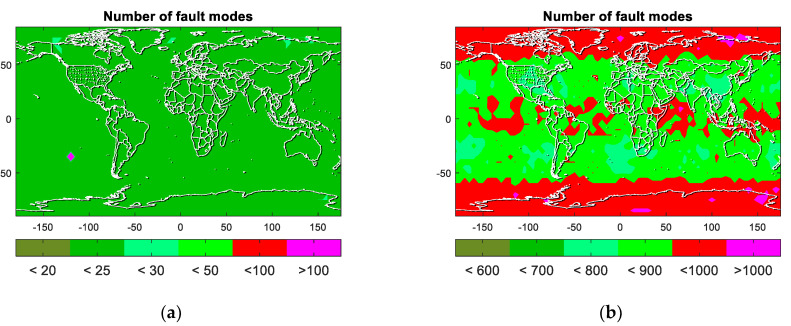
Number of fault modes in ARAIM. (**a**) ARAIM with constellation dynamic selection. (**b**) ARAIM with four constellations.

Figure 6 shows the number of fault modes between ARAIM with constellation dynamic selection and ARAIM with four constellations. Please note that the scales of two figures are not the same. In Figure 6a, ARAIM only needs to monitor fewer than 30 fault modes in most parts of the world. On the other side, the number of fault modes that need to be monitored is larger than 800 using ARAIM with four constellations. It also verified the analysis about the exponential increase in fault subsets in above sections.

**Figure 7:**
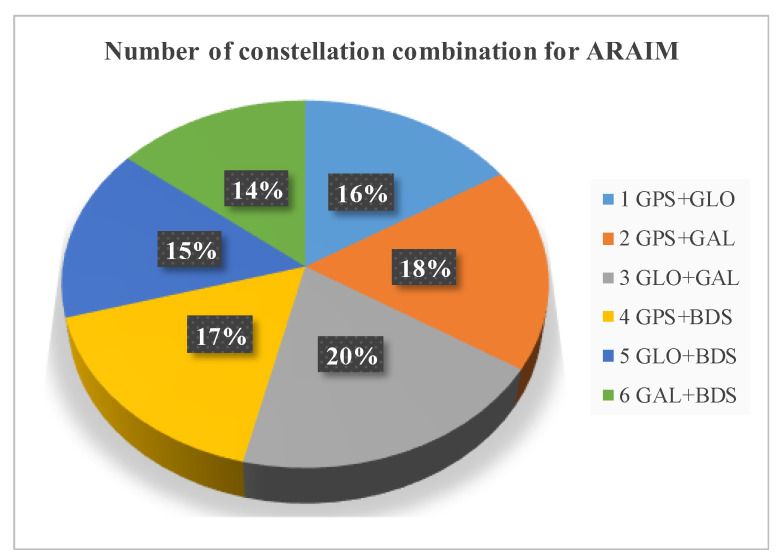
Selection of constellation combination in one sidereal day.

**Figure 8:**
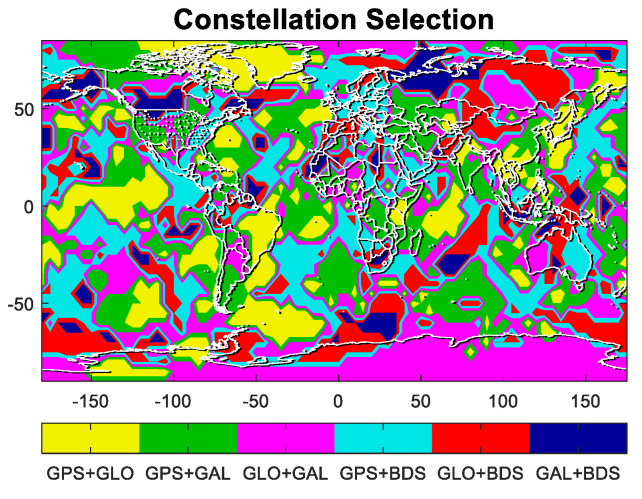
Worldwide distribution of constellation combination.

Last is the distribution of constellation dynamic selection in the whole world. Figure 7 gives the percentages of constellation combination selected in one sidereal day and Figure 8 shows selection results in one random epoch, where six colors represent six constellation combination. The selection is fairly average, no matter from the total number or worldwide distribution. There are, indeed, performance differences of four GNSS constellations in different regions, but this difference is definitely small and relatively random.

#### 4.1.3. Results under Depleted Scenario

As mentioned in the section of introduction, the robustness of ARAIM with two constellations is not enough as the depleted scenarios like satellite outages would hurt the ARAIM availability seriously. To evaluate the robustness of the proposed constellation selection method. The availability under depleted scenario is tested in this section further. A GPS satellite is removed from the constellation to simulate the scenario that one GPS satellite is in maintenance or excluded due to fault. The availability under three above ARAIM modes is tested. The results are shown in Table 4.

As one GPS satellite is removed, in the first two groups, the ARAIM worldwide availability reduces to only about 50% and it is a heavy hit to ARAIM application. The last three groups show good robustness under this circumstance, which are all about 100%. However, the principles to defend one satellite outage are not the same. The ARAIM with four constellation uses the advantage of more visible satellites while the proposed method chooses the best two constellations in spatial vertical performance. The robustness of the proposed ARAIM is no less than that of the ARAIM with four constellations.

### 4.2. Fixed-Point Availability Test

**Figure 9:**
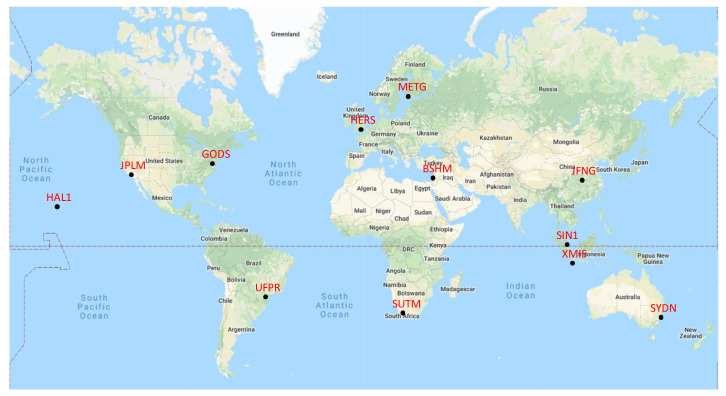
Distribution of observation stations. The black points are station locations, and the red words are station names.

To further prove the superiority of the proposed method, actual fixed-point GNSS signals are collected in this subsection to evaluate the ARAIM availability. The actual GNSS signals are got from the open access high-quality GNSS data with the support of International GNSS Service (IGS) network, including observation files from different stations around the world and the combined multi-GNSS broadcast ephemeris file. Considering the latitudes and continents, observations from 12 stations are chosen for statistical analysis and every station can track GPS, GLONASS, Galileo and BDS. Distribution of the stations chosen are show in Figure 9. The observation data between 2019-July-09 00:00 and 2019-July-09 23:59 are collected for statistics. The data interval is 30 s and the number of valid observations in every station is 2880.

Four ARAIM groups are considered in this test: ‘GPS + Galileo’, ‘GPS + BDS’, four constellations, and constellation dynamic selection. The statistical results are shown in Table 5. The values in one row represent the availability results of different ARAIM modes at one station and the values in one column represent the availability results of one ARAIM mode at different stations. Particularly, the mean availability time is given in the last row. Bold numbers are values beyond 99.5%.

It should be noted that the current GNSS constellations are not all in their nominal configurations. The number of Galileo and BDS MEO satellites have not reach those of their nominal configurations. On the other side, GPS deploys more spare satellites than the need of nominal configuration. It resulted in the availability time varied from about 60% to nearly 100% when fixed dual-constellation is adopted. The ARAIM availability is not evenly spread and rare to reach 99% under the current GNSS constellations. The mean availability results of ‘GPS + Galileo’ and ‘GPS + BDS’ are only 85.76% and 93.00%. However, the availability results under ARAIM with four constellations and ARAIM with constellation dynamic selection are much better than those of ARAIM with two fixed constellations. All values in these two modes exceeded 99% and the results of the latter mode are much close to those of the former one. The mean values are as high as 99.81% and 99.62%, respectively. The proposed constellation dynamic selection method shows strong applicability and generality under the current dynamic developing GNSS constellations. Besides the aerospace application, it is of great help to promote the application of ARAIM in autonomous vehicles and intelligent transportation system.

## 5. Conclusions

From the perspective of integrity risk control of potential hazards uncertainty and user algorithm implementation complexity, the concept of constellation dynamic selection is proposed in this paper. The stable and healthy development of four GNSS constellations and the baseline ARAIM user algorithm based on snapshot principle provide the feasibility to dynamically select different constellations to evaluate the GNSS integrity in different locations and different epochs.

A practical and computationally effective ARAIM method based on constellation dynamic selection is implemented. Two alternative rules are given to select the targeted two constellations with the best vertical geometry performance. Only the two constellations with the minimum VDOP are selected and replaced the other constellations for integrity monitoring. The test results show that the ARAIM worldwide availability can reach 100% under LPV-200 criteria with less than 10% of the computational complexity of ARAIM with four constellations. The proposed method also shows robustness under depleted configurations like satellite outages. Furthermore, the statistics results based on observation stations validated the superiority of the proposed method under current GNSS constellations. A tradeoff is achieved between the high availability and robustness of ARAIM with three or four constellations and the practicability and accessibility of ARAIM with two constellations.

The proposed method is especially suitable for expanding the application of ARAIM technology in harsh environment. We also noticed the criterions for constellation selection can be improved significantly, just as the increasing research for satellite selection. With the further study of satellite/constellation prior fault probability and performance in space, the ISM details of different constellations will definitely be different. How to improve the dynamic selection criterion with the ISM to guarantee the best integrity is the next work.

## Figures and Tables

**Table 1:** Parameters for ARAIM availability evaluation.

Classification	Parameter	Value
LPV-200	VAL	35 m
	HAL	40 m
	EMT	15 m
	*σ_acc_,*req*__*	1.87 m
ISM	*P_const.j_*	10^−5^
	*P_sat_,*i*__*	10^−4^
	*σ_URA,i_*	1 m
	*σ_URE,i_*	2/3*σ_URA_*_,*i*_
	*b_nom_*,*_i_*	0.75 m
Integrity parameters for ARAIM	*P_THRES_*	9 × 10^−8^
	*PHMI_VERT_*	9 × 10^−8^
	*PHMI_HOR_*	1 × 10^−8^
	*P_FA_*	4 × 10^−6^

**Table 2:** ARAIM modes and groups.

No.	Mode	Group by
1	ARAIM with two fixed constellations	GPS + Galileo
2		GPS+ BDS
3	ARAIM with four constellations	GPS + GLONASS + Galileo + BDS
4	ARAIM with constellation dynamic selection	VDOP of constellation combination
5		VDOP of single constellation

**Table 3:** Worldwide availability and relative computational complexity of five groups.

No.	Mode	Group by	Worldwide Availability	Relative Computational Complexity
1.	ARAIM with two fixed constellations	GPS + Galileo	89.01%	7.08%
2.		GPS + BDS	89.08%	7.09%
3.	ARAIM with four constellations	GPS + GLONASS + Galileo + BDS	100%	100%
4.	ARAIM with constellation dynamic selection	VDOP of two constellation combination	99.33%	9.69%
5.		VDOP of single constellation	100%	7.78%

**Table 4:** Worldwide availability of ARAIM in depleted GPS configuration.

No.	Mode	Group by	Worldwide Availability
1.	ARAIM with two fixed constellations	GPS + Galileo	48.82%
2.		GPS + BDS	50.76%
3.	ARAIM with four constellations	GPS + GLONASS + Galileo + BDS	100%
4.	ARAIM with constellation dynamic selection	VDOP of two constellation combination	99.33%
5.		VDOP of single constellation	99.95%

**Table 5:** ARAIM availability statistical results of observation stations.

Number	Station Name	GPS + Galileo Constellations	GPS + BDS Constellations	Four Constellations	Constellation Dynamic Selection
1	HAL1	80.90%	87.88%	100%	99.34%
2	JPLM	86.25%	88.65%	99.34%	99.31%
3	GODS	77.08%	92.36%	99.34%	99.13%
4	UFPR	97.05%	68.75%	100%	99.86%
5	HERS	90.76%	97.19%	99.24%	99.24%
6	METG	92.71%	98.54%	100%	100%
7	BSHM	88.75%	97.05%	100%	100%
8	SUTM	96.60%	97.95%	100%	99.31%
9	SIN1	89.30%	99.06%	100%	99.79%
10	XMIS	91.84%	97.85%	100%	100%
11	JFNG	61.46%	98.89%	100%	99.65%
12	SYDN	76.60%	91.81%	99.79%	99.76%
Mean value		86.06%	92.97%	99.99%	99.62%
